# Serine racemase: a key player in apoptosis and necrosis

**DOI:** 10.3389/fnsyn.2014.00009

**Published:** 2014-04-21

**Authors:** Nadia Canu, Maria Teresa Ciotti, Loredano Pollegioni

**Affiliations:** ^1^Dipartimento di Medicina dei Sistemi, Università degli Studi di RomaRoma, Italy; ^2^Istituto di Biologia Cellulare e Neurobiologia, Consiglio Nazionale delle RicercheRoma, Italy; ^3^Dipartimento di Biotecnologie e Scienze della Vita, Università degli Studi dell'InsubriaVarese, Italy; ^4^Centro Interuniversitario di Ricerca in Biotecnologie Proteiche “The Protein Factory,” Politecnico di Milano, ICRM-CNR Milano and Università degli studi dell'InsubriaMilano, Italy

**Keywords:** serine racemase, D-serine, neurodegeneration, apoptosis-necrosis shift, NMDAR, neurological disorders, review

## Abstract

A fine balance between cell survival and cell death is required to sculpt the nervous system during development. However, an excess of cell death can occur following trauma, exposure to neurotoxins or alcohol, and some developmental and neurodegenerative diseases, such as Alzheimer's disease (AD). N-Methyl-D-aspartate receptors (NMDARs) support synaptic plasticity and survival of many neuronal populations whereas inappropriate activation may promote various forms of cell death, apoptosis, and necrosis representing the two extremes of a continuum of cell death processes both “*in vitro*” and “*in vivo*.” Hence, by identifying the switches controlling pro-survival vs. apoptosis and apoptosis vs. pro-excitotoxic outcome of NMDAR stimulation, NMDAR modulators could be developed that selectively block the cell death enhancing pro-survival signaling or synaptic plasticity mediated by NMDAR. Among these modulators, a role is emerging for the enzyme serine racemase (SR) that synthesizes D-serine, a key co-agonist with glutamate at NMDAR. This review summarizes the experimental evidence from “*in vitro*” neuronal cultures—with special emphasis on cerebellar granule neurons (CGNs)—and “*in vivo*” models of neurodegeneration, where the dual role of the SR/D-serine pathway as a master regulator of apoptosis and the apoptosis-necrosis shift will be discussed.

## Introduction

Cell death is an obligatory process in the development, maintenance, and plasticity of the nervous system, and both anti-death and pro-death modulators are key elements in designing neural architecture. Conversely, the dysregulation of mechanisms controlling cell death machinery activation can cause developmental, neoplastic, and neurodegenerative disorders.

Cell death can manifest in many forms, which can be distinguished by various histological criteria “… (apoptosis, necrosis, autophagy), enzymological criteria (with and without the involvement of nucleases or of distinct classes of proteases, such as caspases, calpains, cathepsins, and transglutaminases), functional aspects (programmed or accidental, physiological or pathological) or immunological characteristics (immunogenic or non-immunogenic)….” (Kroemer and Galluzzi, 2009). However, in many pathological conditions and in *in vitro* and *in vivo* disease models, neuronal demise may result from complex, intersecting, and merging fatal pathways (Jellinger, 2001; Andorfer et al., 2005; Canu et al., 2005) that often imply the shift of one particular mode of cell death to another (e.g., apoptosis/necrosis or necrosis/apoptosis shift), possibly via intermediate types of cell death. Such complexity holds implications for the subsequent fate of the tissue because inhibiting a particular mechanism renders the brain vulnerable to alternative death modes (Puyal et al., 2013). Hence, by identifying switches between different types of cell death modulators able to block selectively a specific death pathway without causing the concomitant emergence of alternative pathways could be developed.

Death signals are spatially and temporally segregated in neurons, for example, at remote synaptic sites (Mattson et al., 1988; Berliocchi et al., 2005). Indeed, much of the biochemical machinery involved in apoptosis can be activated in synaptic terminals, where it can remodel synapses or alter synaptic function and promote localized degeneration of synapses and neurites under both physiological and pathological conditions. For example, caspase-3 is crucially involved in monitoring, locally, protein levels in retinal growth cone formation (Campbell and Holt, 2003), and NMDAR-dependent caspase-3 activity is required for memory storage in long-term depression (LTD) and AMPA receptor internalization in hippocampal neurons (Li et al., 2010). In a similar way, the ubiquitin–proteasome system (UPS) is implicated in apoptosis (Canu et al., 2000; Sun et al., 2004), synaptic strength, homeostatic plasticity, axon guidance, and neurite outgrowth (Hamilton and Zito, 2013).

The relevance of spatially and temporally segregated death programs has also been confirmed by studies in neurodegenerative models, where a stage of synaptic dysfunction (for example, electrophysiological deficits), microanatomical changes (such as neurite retraction and synapse loss) (D'Amelio et al., 2011), and cognitive deficits may precede neurodegeneration. Hence, early perturbation of synapse integrity or function has been suggested to be even more relevant than late neuronal loss in slow degenerative disorders (reviewed in Gillingwater and Wishart, 2013), such as Alzheimer (AD) (Davies et al., 1987; Sze et al., 1997; Hatanpää et al., 1999; Mota et al., 2014); Huntington (HD) (Mangiarini et al., 1996; Yamamoto et al., 2000), and Parkinson diseases (PD) (Paumier et al., 2013) or in psychiatric disorders such as schizophrenia (Faludi and Mirnics, 2011) where neuronal loss is subtle, thus suggesting that loss of neurites and synaptic dysfunction may define the hystopathological phenotype of AD, HD, PD, or schizophrenia.

Death programs are activated with extraordinarily reproducible patterns in specific nuclei and with specific frequencies at particular times of nervous system development. However, they may also be inappropriately activated by various insults, such as trophic factor withdrawal, altered NMDAR stimulation, excitotoxicity, misfolded proteins, reactive oxygen and nitrogen species, mitochondrial-complex inhibition, calcium entry, death-receptor activation, etc.

Here, NMDAR represents the main neuronal, specific signaling system that bidirectionally regulates cell fate by stimulating pro-survival or pro-death signaling; the latter share many common intracellular signal pathways with NMDAR-dependent, long-term potentiation (LTP) and LTD, respectively (Bartlett and Wang, 2013). NMDAR may decide whether, when, and how neurons die. Both hypofunction and overstimulation of NMDAR can cause cell death. Blockade of NMDAR elicits apoptosis, while overstimulation of NMDAR can trigger either apoptosis or necrosis, depending on the intensity of receptor activation (Bonfoco et al., 1995; Staton and Bristow, 1997). Precisely, short exposure to low concentrations of glutamate or NMDA evokes apoptosis in cortical neurons (Leist et al., 1999), whereas intense exposure to high concentrations of NMDA or glutamate induces necrotic cell damage (Bano et al., 2005). In this latter case, the degree of cell loss depends on the magnitude and duration of synaptic and extrasynaptic NMDAR coactivation (Zhou et al., 2013).

Unlike other neurotransmitter receptors, the simultaneous binding of two co-agonists, glutamate and glycine or D-serine, with different biophysical properties of ion permeation is required to activate NMDAR (Johnson and Ascher, 1990). The continuous (i.e., nonsynaptically released) presence of D-serine or glycine is an absolute prerequisite for both NMDAR activity during normal neurotransmission and NMDAR overstimulation that occurs in various neurological disorders (Kleckner and Dingledine, 1988; Danysz and Parsons, 1998).

**Figure 1:**
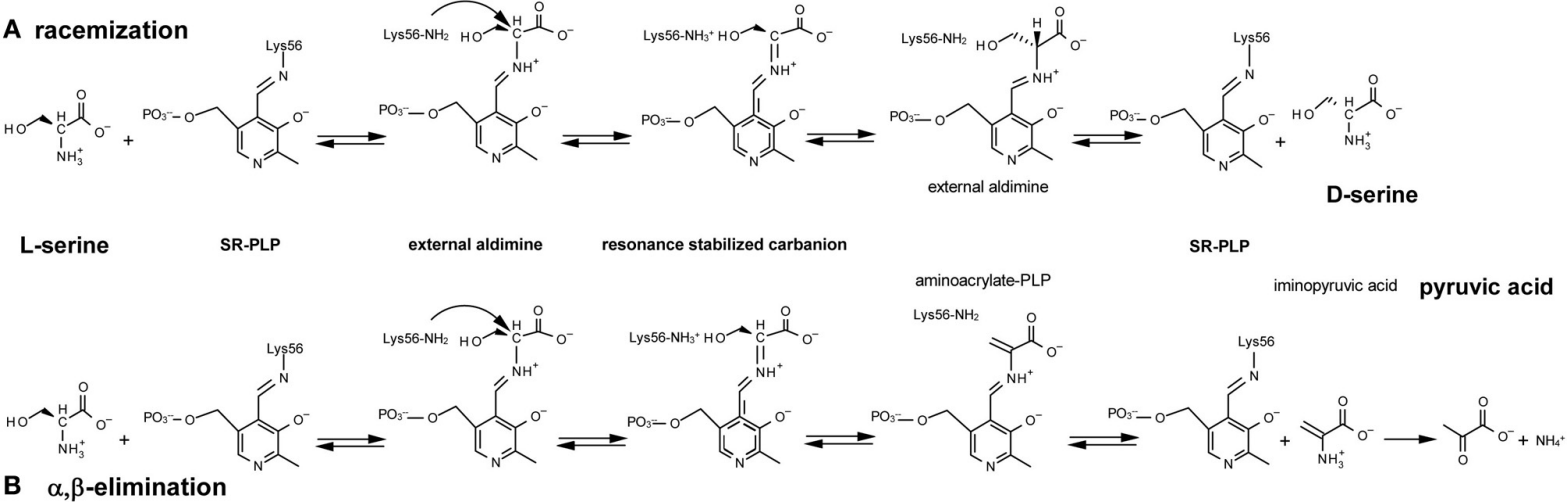
**Reactions catalyzed by serine racemase: (A) racemization; (B) α, β-elimination.** Lys56 of SR binds the PLP cofactor, forming an internal aldimine; then SR reacts with L-serine to yield an external aldimine; α-proton abstraction from this intermediate gives a resonance-stabilized carbanion. Two alternative pathways are possible starting from this intermediate (see text for details).

D-serine is synthesized from L-serine by glial and neuronal enzyme serine racemase (SR, EC 5.1.1.18) (De Miranda et al., 2002) (Figure 1) and is selectively degraded by both SR and the peroxisomal D-amino acid oxidase (DAAO, EC 1.4.3.3) (Sacchi et al., 2012). Thus, it is not surprising that D-serine and the enzymes involved in its metabolism are crucially involved in several physiological and pathological processes related to NMDAR function and dysfunction.

The present review focuses on how activation of a death program implies that the SR-D-serine pathway is modulated and how this, in turn, contributes to the cell death phenotype. Firstly, the role of NMDAR on survival and cell death will be briefly reviewed. Secondly, the biology and regulation of SR will be widely analyzed. Thirdly, results—from a cerebellar granule neuron (CGN) *in vitro* model of apoptosis—suggesting that the SR-D-serine pathway can be modulated by apoptosis to execute the death program and to channel neurons to a necrotic phenotype will be presented and discussed. Finally, different disease-related brain dysfunctions associated with altered SR expression will be presented and discussed in relation to death program activation and altered NMDAR activity.

## NMDA receptors

Normal physiological brain function and neuronal survival require adequate activation of NMDARs. These receptors are glutamate-gated ion channels composed of different subunits with differing biophysical and pharmacological properties, interacting partners, and subcellular localization (Paoletti et al., 2013; Sanz-Clemente et al., 2013). NMDAR subunits are classified into three subfamilies: one NR1 subunit (constituted by 8 splice variants), four NR2 subunits (2A, 2B, 2C, and 2D), and two NR3 subunits (3A and 3B) (Traynelis et al., 2010). All NMDARs comprise two NR1 subunits and two copies of NR2 and/or NR3 subunits: the latter two copies can be identical or different, thus yielding di-heteromeric and tri-heteromeric receptors, respectively, which may be present in various areas of neuronal plasma membrane (synaptic and extrasynaptic).

Molecular mechanisms controlling subunit-specific NMDAR function include developmental regulation of subunit transcription/translation, differential trafficking through the secretory pathway, post-transcriptional modifications (i.e., phosphorylation), and protein-protein interactions (Sanz-Clemente et al., 2013). Due to the early expression in development, the N2B, 2D, and N3A subunits are important for synaptogenesis and synaptic maturation (Henson et al., 2010), while in the adult forebrain, synaptic NMDARs are largely di-heteromeric NR1/NR2A and tri-heteromeric NR1/NR2A/NR2B receptors. Peri- and extrasynaptic sites are enriched in NR2B-containing receptors (Hardingham and Bading, 2010).

NMDAR subunits consist of four domains: two large globular bilobate domains in the extracellular region, comprising the N-terminal domain (which is involved in subunit assembly and allosteric regulation) and the agonist-binding domain (which binds the primary agonist glutamate in NR2 subunits and coagonist glycine or D-serine in NR1 and NR3 subunits); the transmembrane domain composed of three transmembrane helices and a pore loop that lines the ion selectivity filter; and a long intracellular C-terminal domain (which is involved in receptor trafficking, anchoring, and coupling to signaling molecules); reviewed in Paoletti et al. (2013).

The subunit composition of NMDARs is modified during development in response to neuronal activity or sensory experiences (Henson et al., 2010); plasticity can also develop at adult synapses. Various mechanisms may be responsible for the NR2B-to-NR2A subunit modification: (i) novel receptors are inserted through trafficking from the endoplasmic reticulum; (ii) existing synaptic receptors are removed through endocytosis; and (iii) clearance of fast-moving, NR2B-containing receptors from synaptic sites through lateral diffusion (Groc et al., 2006). Owing to the mobility of NMDAR between synaptic and extrasynaptic sites, receptor number and subunit composition are finely regulated.

Using specific enzymes that degrade D-serine or glycine—*Rhodotorula gracilis* D-amino acid oxidase or *Bacillus subtilis* glycine oxidase (Pollegioni et al., 1993; Fantinato et al., 2001; Job et al., 2002)—and electrophysiological recordings in the CA1 region of the hippocampus (largely populated by NR2A receptors which have a better affinity for D-serine than for glycine), evidence was provided that D-serine is the coagonist at synaptic NMDARs, whereas glycine is the coagonist at extrasynaptic NMDARs (Papouin et al., 2012). This functional compartmentalization arises from glycine and D-serine availability and matches the preferential affinity of NMDARs for these two coagonists. Indeed, glycine strongly and rapidly inhibits the mobility of NR2A-NMDARs, whereas D-serine preferentially slows down the lateral diffusion of NR2B-containing receptors. Finally, it was demonstrated that LTP and NMDA-mediated excitotoxicity depend on synaptic NMDARs, whereas LTD requires both synaptic and extrasynaptic receptors.

A weakening in memory function with aging is often linked to impaired synaptic plasticity, which might arise from altered function and/or expression of NMDARs (i.e., the NR2B subunit): recovery of NMDAR decline is known to improve LTP (Magnusson et al., 2010). The recent recognition that NMDAR subunit composition can be remodeled according to activity, even in adults, further supports the notion that the molecular composition of the receptor is tailored to match the needs of specific neural functions.

## NMDAR in survival and cell death

Both NMDAR hypofunction and NMDAR hyperactivity (excessive Ca^2+^ influx through NMDARs) induce neuronal death and are deleterious (Sanz-Clemente et al., 2013).

The first hint that NMDAR supports neuronal survival came from the findings that cultured rat CGNs survived in the presence of depolarizing concentrations of KCl (Gallo et al., 1987). Given that this condition was suggested to mirror the afferent glutammatergic inputs received by the CGNs *in vivo* (Borsello et al., 2000), it was hypothesized that NMDAR signaling ensures survival of newly generated granule neurons. It was then shown that NMDAR activation by exogenous NMDA prevented rat CGN survival from decreasing under low KCl concentrations (Balázs et al., 1988, 1989) by inhibiting programmed cell death (PCD) induced by low potassium levels (Yan et al., 1994; Alavez et al., 2006; Xifró et al., 2006; Bazán-Peregrino et al., 2007; Jantas and Lason, 2009; Esposito et al., 2012). Then, it was reported that NMDA offered protection to other neuronal populations *in vitro*, such as rat cortical neurons, subjected to several apoptotic stimuli, including serum deprivation and treatment with NMDAR antagonists or PI-3 kinase inhibitors (Hetman et al., 2000; Terro et al., 2000). Similarly, NMDAR antagonists elicited apoptosis in cortical and retinal neurons in culture (Hwang et al., 1999; Takadera et al., 1999), worsened rat cortical neuron apoptosis induced by serum deprivation (Hetman et al., 2000; Terro et al., 2000), and caused a marked decrease in the number of dopaminergic neurons in P2-P3 rat midbrain slice (Katsuki et al., 2003). Transient blockade of the NMDAR during prenatal or early neonatal life has been shown to trigger death in multiple developing forebrain structures (Gould et al., 1994; Ikonomidou et al., 1999). These findings were also confirmed in primates, where prenatal blockade of NMDAR by antagonists, anesthetics, or substances of abuse compromise neuronal survival during brain development.

The apoptotic responses to decreased NMDAR activity during the critical period of synapse formation suggests that NMDAR supports survival of those neurons that have established appropriate synaptic connections (Adams et al., 2004). Thereafter, the contribution of NMDAR to survival decreases. Indeed, NMDAR hypofunction or blockade in adult brain does not cause cell death, but rather damages the synapses, for example, by altering NMDAR subunit trafficking into dendritic spines (Aoki et al., 2003), decreasing dendritic spine density (Velázquez-Zamora et al., 2011), or impairing function and altering the morphology of dendritic spines, as in medium spiny neurons of corticostriatal slices treated with NR2A antagonist (Vastagh et al., 2012), which renders neurons more vulnerable to neurodegeneration.

On the other hand, overactivation of NMDARs reduces brain cell survival signals and disrupts brain function, causing neurotoxicity through calcium dysregulation and oxidative stress. Hyperactivation of NMDAR is a major cause of cell death following acute neuronal injury, such as stroke and trauma, and is also implicated in neurodegenerative diseases, such as PD, HD, and late AD (Hardingham and Bading, 2003).

**Figure 2:**
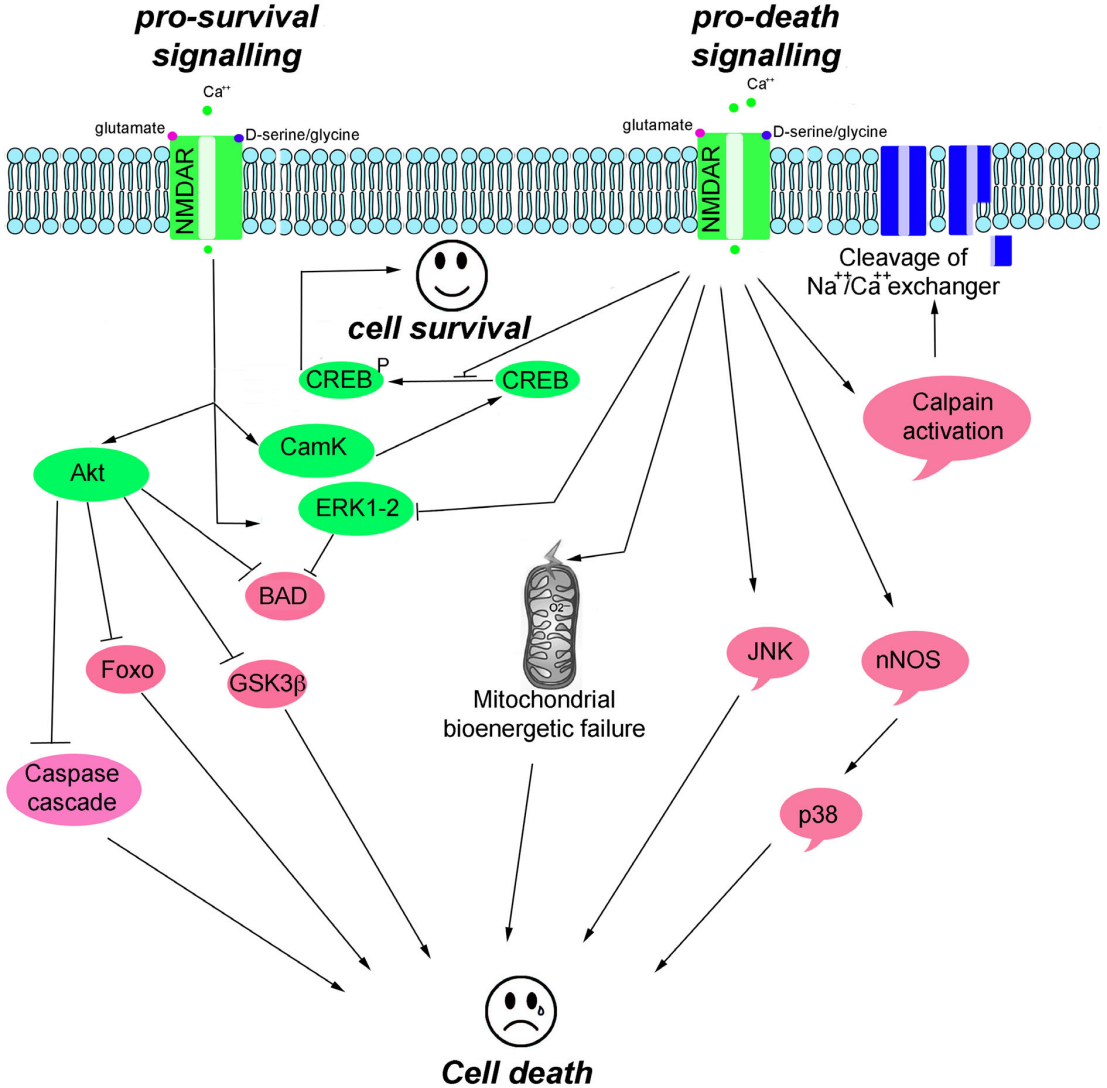
**Scheme illustrating the main pro-survival and pro-death signals triggered by NMDAR activity**.

NMDAR-mediated pro-survival activity is, in part, mediated through the suppression of pro-apoptotic kinases, such as glycogen synthase kinase 3 beta (GSK-3β), and the activation of survival signaling kinases, including the extracellular signal-regulated kinases (ERK1/2), the serine/threonine protein kinase B (Akt, also known as PKB), and Ca^2+^/calmodulin-dependent protein kinase II/IV (CamKII/IV) that converge on cAMP responsive element-binding protein (CREB) to activate pro-survival genes (Hetman and Kharebava, 2006). NMDAR-mediated toxicity triggers an excessive entry of Ca^2+^, which initiates a series of cytoplasmic and nuclear processes that promote neuronal cell death, such as CREB shut-off signaling; activation of Ca^2+^-activated proteolytic enzymes (e.g., calpains that degrade cytoskeleton proteins and Na^+^Ca^2+^ channel exchanger); and increased levels of phospho c-Jun N-terminal kinases (pJNK), phospho p38 kinase, and nitric oxide synthase (NOS) (Figure 2).

## Serine racemase

### General properties

Mammalian SR is a pyridoxal-5′ phosphate (PLP)-containing enzyme that was isolated at the end of the last millennium from rodent brain by Snyder's group (Wolosker et al., 1999). SR was identified for its ability to catalyze the racemization of L-serine to D-serine (Figure 1A) although, subsequently, SR has been shown to catalyze α,β-elimination of water from L- or D-serine to yield pyruvate and ammonia (Figure 1B) (De Miranda et al., 2002; Foltyn et al., 2005; Pollegioni and Sacchi, 2010; Campanini et al., 2013).

The first observation that SR catalyzes an α,β-elimination reaction with L-serine was demonstrated in cells transfected with SR since they produced high levels of pyruvate along with D-serine (De Miranda et al., 2002): the pyruvate/D-serine ratio was ≈4, indicating that the α,β-elimination reaction predominates over the racemization. In contrast, little or no pyruvate was produced from L-serine by preparing purified SR: the recombinant enzyme lacks essential cofactor(s), later on identified as Mg-ATP. Since ADP is also equally effective in SR activation, it was argued that the role of ATP is not related to an energy requirement for enzyme activity.

Both the racemization and elimination reactions from L-serine were activated up to 10-fold by >10 μM MgCl_2_/CaCl_2_ and by >100 μM ATP (interestingly, Mg^2+^ and ATP modulate SR activity at concentrations below those found in the cytosol) (Cook et al., 2002; De Miranda et al., 2002). Mg^2+^ or Ca^2+^ cations interact with similar affinity to a specific site: the former should represent the physiological ligand since the free Mg^2+^ concentration in the cells is several orders of magnitude higher than Ca^2+^. The racemase activity was independently stimulated by both Mg^2+^ and ATP: the nucleotide increased SR activity even in the presence of EDTA, and the effect due to divalent ion and ATP was additive (De Miranda et al., 2002). In the presence of 1 mM ATP, the K_m_ for L-serine is decreased 10-fold with little change in V_max_ (Neidle and Dunlop, 2002). EDTA impairs the α,β-elimination reaction, which is instead stimulated by 100 μM Mg^2+^ in the absence of exogenous ATP or by 10 μM of divalent ion in the presence of the nucleotide (De Miranda et al., 2002; Foltyn et al., 2005). Very recently, Mozzarelli's group demonstrated a cross-talk between the allosteric and active sites in SR, leading to the stabilization of two alternative protein conformations with ATP affinities of ~10 μM and 1.8 mM. In fact, the dependence of α,β-elimination and L-serine racemization activities on ATP concentration was strongly cooperative (Hill coefficients ~2), as was ATP binding to the holoenzyme. The active site ligand glycine increased the SR affinity for ATP by 22-fold and abolished cooperativity while ATP increased the noncooperative glycine binding 15-fold (Campanini et al., 2013; Marchetti et al., 2013).

A review of the morphology, gene expression, neurotransmission/neurodegeneration, and behavior of SR knockout mice was recently published (Wolosker and Mori, 2012).

### Catalytic mechanism

**Figure 3:**
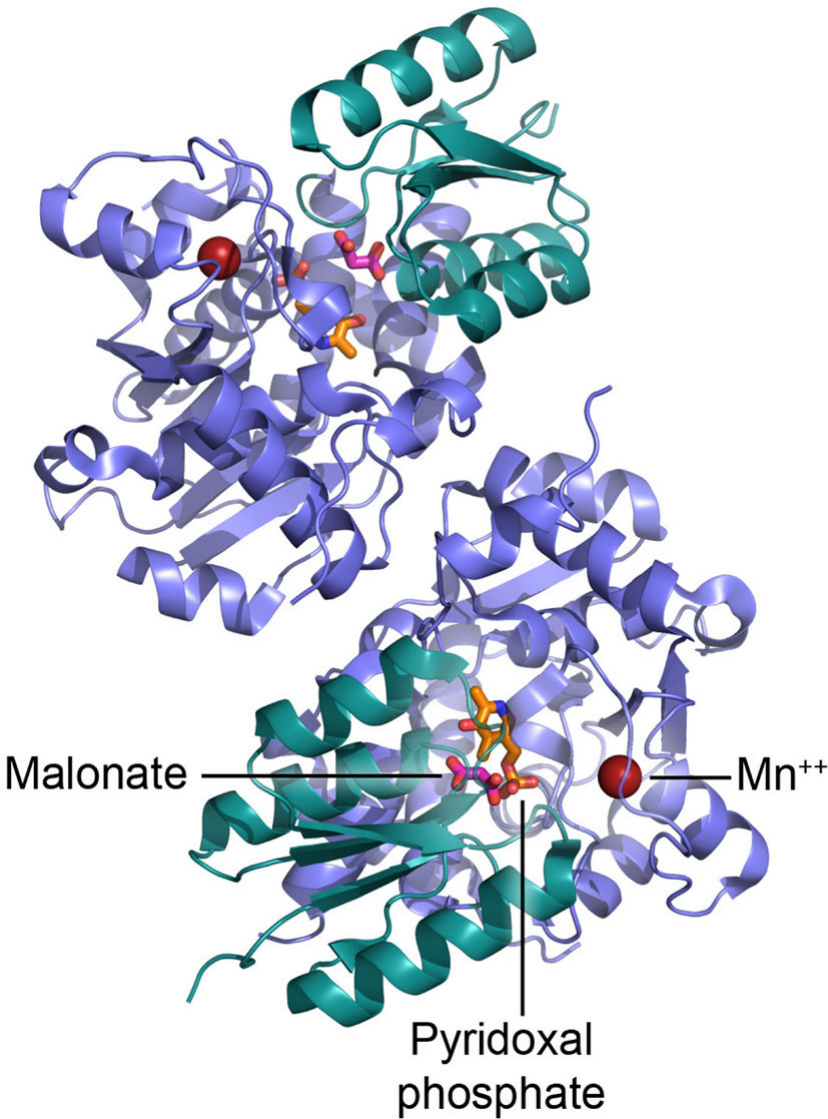
**Dimeric structure of human SR and details of the active site.** PLP is shown in orange, malonate in red, and Mn^2+^ as red sphere.

Sequence and structural analyses indicate that SR belongs to the fold type II group of PLP-dependent enzymes, together with many other racemases and dehydratases. The recent determination of the crystal structure of the *Saccharomyces pombe* homolog (pdb code 1WTC) (Goto et al., 2009) followed by the rat and human SR (pdb code 3L6B and 3HMK, respectively) (Smith et al., 2010) has given us a deeper understanding of SR catalytic mechanism and the location of the cofactor binding sites (Figure 3). The enzyme is a homodimer, each monomer (340 amino acids) consisting of two domains, a small and a large domain connected by a flexible loop (Goto et al., 2009; Smith et al., 2010). The large domain contains most of the residues that interact with PLP (Lys56 that forms the Schiff base with PLP during catalysis is conserved in PLP-containing racemases) and the residues involved in the dimerization; the site for binding of Mg-ATP is located at the interface between the domains, outside the catalytic site (Figure 3).

It has been proposed that binding of L-serine to SR in complex with Mg-ATP alters the enzyme conformation from an open to a closed form (Goto et al., 2009). During this reorientation of the small and large domains, the small domain is likely to reposition itself toward the catalytic site and orient Ser84 toward the substrate (Smith et al., 2010). Mg^2+^ is coordinated by the carboxylic groups of Glu210 and Asp216 and the backbone carbonyl oxygen of Ala214 (Goto et al., 2009; Smith et al., 2010).

Mechanicistically, the racemization and α,β-elimination reactions share the same intermediate, represented by a resonance-stabilized carbanion (Figure 1): this intermediate forms a partition between the two pathways. L-Serine binds to PLP, yielding an external aldimine intermediate, followed by the abstraction of the α-proton by Lys56 and formation of a planar resonance-stabilized carbanion (Foltyn et al., 2005). At this point, protonation in the opposite side of the carbanion intermediate (mediated by the Ser84-OH group) (Goto et al., 2009; Smith et al., 2010) generates D-serine (racemization reaction). Since the proton abstraction and the reprotonation steps are performed by different residues (Lys56 and Ser84) that work as acid/base catalysts, SR racemization is consistent with a two-base mechanism. In the α,β-elimination reaction, protonation of the β-hydroxy group of the substrate eliminates water from the carbanion intermediate and an unstable aminoacrylate intermediate is formed, followed by release of iminopyruvate and spontaneous hydrolysis into pyruvate and NH^4+^ (Figure 1). The partition between the elimination of the β-hydroxyl group and racemization (reprotonation on αC) is affected by Mg-ATP, which favors the former (Foltyn et al., 2005).

Mouse SR activity is inhibited by amino acids containing –SH groups (cysteine and homocysteine) or electron-withdrawing groups on the β-carbon of alanine (β-haloalanine): these compounds react with PLP to yield thiazolidine derivatives. Most interestingly, L-aspartate and glycine are also competitive inhibitors of SR (K_i_ is 1.9 and 0.15 mM, respectively), indicating that their *in vivo* concentration plays a role in D-serine synthesis (i.e., glycine concentration in astrocytes is in the 3- to 6-mM range) (Dunlop and Neidle, 2005). Several dicarboxylic acids are strong, competitive inhibitors of SR (Strísovský et al., 2005), but so far, no selective enzyme inhibitors have been discovered. The most potent known SR inhibitor is L-erythro-3-hydroxyaspartate (K_i_ = 49 μM), which competes with L-serine (Strísovský et al., 2005).

### Physiological role of α,β-elimination

The physiological role of SR is probably different in different tissues. In the liver, a tissue lacking NMDAR, pyruvate generated by the α,β-elimination reaction catalyzed by SR should play a metabolic role: SR is located in neuronal processes, where it might significantly contribute to the metabolic demands (Kartvelishvily et al., 2006). On the other end, the SR-catalyzed α,β-elimination reaction provides an unusual mechanism for controlling intracellular D-serine levels in the brain, particularly in the forebrain, where SR expression is highest and the D-serine catabolic enzyme DAAO is poorly expressed.

In neurons, the steady-state levels of D-serine are largely modulated by SR activity: D-serine is produced from L-serine by the isomerization reaction and is also constantly consumed by the α,β-elimination reaction with a rate fast enough to limit the achievable D-serine concentration *in vitro* and in intact cells (Foltyn et al., 2005). For mouse SR, the elimination reaction starting from L-serine possesses a 2-fold higher kinetic efficiency than the racemization reaction (the V^max^/K_m_ ratio is 20 and 12 mM^−1^ min^−1^, respectively) and the racemization of D-serine into L-isomer is 3-fold higher than for the pyruvate elimination reaction (Strísovský et al., 2005; Table 1 in Pollegioni and Sacchi, 2010). The presence of Mg-ATP stimulates D-serine degradation as L-serine is consumed by the purified SR: under equilibrium conditions, almost all L- and D-serine will be converted to pyruvate and ammonia. The impact of α,β-elimination is best appreciated when SR is exposed to a physiological L-serine/D-serine ratio (1 and 0.3 mM of L- and D-serine, respectively): the level of both enantiomers decreases. In constrast, EDTA chelation of divalent ions stabilizes D-serine by blocking the α,β-elimination activity.

A list of the molecules known to modulate the activity of SR is reported in Table 1.

**Table 1:** **Known modulators of SR (activity and stability)**.

**POSITIVE EFFECTS**		
MgCl_2_/MnCl_2_	Increase in activity at >10 μM	De Miranda et al., 2002
ATP	Increase in activity at >100 μM; change in K_m_ for L-serine	De Miranda et al., 2002; Neidle and Dunlop, 2002
	Increase in glycine binding	Marchetti et al., 2013
Glycine	Increase in ATP affinity	Marchetti et al., 2013
Grip1	Activation by interaction through PDZ domain	Kim et al., 2005; Baumgart et al., 2007
PICK1	Activation by interaction through PDZ domain	Hikida et al., 2008
Golga3	Protection from protein degradation	Dumin et al., 2006
**NEGATIVE EFFECTS**		
Cysteine, homocysteine, β-haloalanine	Inactivation by PLP modification	Dunlop and Neidle, 2005
L-aspartate	Competitive inhibitor, K_i_ = 19 mM	Dunlop and Neidle, 2005
Glycine	Competitive inhibitor, K_i_ = 0.15 mM	Dunlop and Neidle, 2005
L-herythro-3-hydroxyaspartate	L-serine competitive inhibitor, K_i_ = 0.049 mM	Strísovský et al., 2005
EDTA	Metal chelation, block of α,β-elimination	Strísovský et al., 2005
Phosphotidylinosityl lipids	Inhibition by palmitoylation and phosphorylation	Balan et al., 2009
Nitric oxide	Inhibition by nitrosylation (reversed by D-serine)	Shoji et al., 2006a

### Physiological regulation of SR

SR is regulated by Grip1 (glutamate receptor interacting protein, usually coupled to the Glu2/3 subunits of the α-amino-3-hydroxy-5-methylisooxazole-4-propionic acid—AMPA-Ca^2+^ channel), which binds to the C-terminal region via its PDZ6 domain (Kim et al., 2005). Both mouse and human SR contain a ValSerCys sequence at their C-terminus, a motif resembling the type II consensus sequence for binding to PSD95/disc large/ZO-1 (PDZ) domains: SR is activated by binding to the PDZ6 domain of Grip1. Full activation of SR requires binding to the remaining part of the C-terminal region of GRIP (Baumgart et al., 2007).

SR also binds to the protein interacting with C kinase 1 (PICK1), a PDZ domain-containing protein proposed to regulate subcellular localization and membrane expression of various binding partners because of the interaction with lipid membranes. Neonatal PICK1-deficient mice display 30% lower D-serine levels in the forebrain, and HEK293 cells transfected with SR or PICK1 (or both) show an increase in D-serine concentration (Hikida et al., 2008).

SR is also modulated by Golga3, the Golgin subfamily A member 3 that is associated with the cytosolic face of the Golgi apparatus. SR is degraded through the UPS, and association of the N-terminal 66 residues of SR with Golga3 decreases SR protein degradation and increases D-serine synthesis (Dumin et al., 2006). Golga3 and SR colocalize in the cytosol perinuclear Golgi region and in neuronal and glia processes in primary cultures; SR strongly bound to the membrane constitutes a pool of available SR to be exported/targeted to different cell compartments (Dumin et al., 2006).

Interestingly, SR is regulated by glutamate receptors via multiple mechanisms by modulating SR interaction with membranes. Binding of SR to the membrane is regulated by NMDAR activation and interaction with phosphatidylinositol lipids (Balan et al., 2009; Mustafa et al., 2009). In primary rat neuronal cultures, NMDAR activation promotes translocation of SR from the cytosol to dendritic membranes, a process that dramatically decreases SR activity and thus D-serine synthesis. Such a translocation requires palmitoylation of SR at the serine/threonine residues and the phosphorylation at Thr227 (Balan et al., 2009). This inhibition of SR should represent a reliable strategy to prevent NMDAR overactivation in vicinal cells or synapses. Indeed, SR is also inhibited via the glial cell membrane by phosphatidylinositol(4,5)-bisphosphate (PIP2) (Mustafa et al., 2009). Degradation of PIP2 by metabotropic glutamate receptor activation releases SR from the membrane and thus SR recovers the activity (Mustafa et al., 2009).

By using the U87 human glioblastoma cell line, SR activity was also demonstrated to be inversely regulated by D-serine and nitric oxide: SR activity was enhanced in a dose-dependent manner by D-serine and was inhibited by NO (Shoji et al., 2006a). Indeed, D-serine induces the denitrosylation of SR. Interestingly, DAAO activity was enhanced by NO in a dose-dependent manner (Shoji et al., 2006b). It was suggested that NMDAR-mediated Ca^2+^ influx at postsynaptic neurons involves Ca^2+^/calmodulin-dependent activation of neuronal NO synthase: the NO produced here diffuses into adjacent astrocytes or neurons to nitrosylate and inhibit SR and activate DAAO (Shoji et al., 2006a,b). Cys113, identified as the target residue of SR nitrosylation, is in close proximity to the ATP-binding region and thus nitrosylation might displace ATP from its binding site and inactivate SR: ATP and NO reciprocally activate and inhibit the enzyme by acting at the same protein site (Mustafa et al., 2007).

A list of the molecules known to modulate the activity or stability of SR is reported in Table 1.

### Localization

Human SR mRNA was mainly identified in brain, heart, skeletal muscle, kidney, and liver tissues (Xia et al., 2004; Yamada et al., 2005). It mainly localized in brain areas containing high levels of endogenous D-serine (i.e., hippocampus and corpus callosum) with intermediate levels in substantia nigra and caudate and negligible levels in brainstem. Multiple protein bands of human SR were observed by Western blot analysis: a 39-kDa band was apparent in HEK293-transfected cells, a band at 42 kDa in human brain extracts, and a band at 62 kDa in kidney and heart, where an additional 80-kDa band was also apparent (Xia et al., 2004). SR was initially localized in astrocytes while subsequent studies showed a predominantly neuronal localization; neurons represent the cells from which D-serine is released upon membrane depolarization (Kartvelishvily et al., 2006; Wolosker et al., 2008).

During postnatal development in the brain, SR is localized in glutamatergic neurons in the cerebral cortex and in the glutamatergic pyramidal neurons of the hippocampus, where it is mainly present at postsynaptic sites. In the adult cerebellum, levels of SR expression are lower than those reported for the telencephalic regions; a weak SR expression was also apparent in GABAergic Purkinje cells (Miya et al., 2008). These findings suggest that SR is expressed in principal neurons of given neuronal regions, irrespective of the excitatory or inhibitory signature. Furthermore, SR was also identified in samples from the perireticular nucleus, a transient structure of developing brain in humans, pointing to the importance of SR function for developing correctly formed corticothalamic and thalamocortical connections by stimulating D-serine-dependent NMDAR activity.

It is noteworthy that synthesis of both neuronal and astrocytic D-serine in the brain is dependent on 3-phosphoglycerate dehydrogenase, an enzyme that occurs mainly in astrocytes and which catalyzes the first step in L-serine biosynthesis: L-serine shuttles from astrocytes into neurons where it is transformed by neuronal SR to D-serine (Ehmsen et al., 2013).

## The serine racemase-D-serine-related pathway in neuronal apoptosis

Although our knowledge of the mechanisms underlying neuronal cell death is far from complete, research on rodent and human models has identified factors regulating NMDAR activation and signaling as being a component of neuronal demise.

Apoptosis, a type of PCD, is a genetically directed process that involves a controlled sequence of steps in which cells signal self-destruction. It is activated either by the presence of a stimulus or removal of a suppressing agent or stimulus and constitutes a normal physiological process of eliminating DNA-damaged, superfluous, or unwanted cells without damaging neighboring cells or eliciting an inflammatory response. Such a complex process, which occurs in an orderly manner, is characterized by specific biochemical (caspase and calpain activation) and morphological events (DNA fragmentation, neurite retraction, and membrane blebbing). It depends on the activity of an integrated network of genes, regulated at both the transcriptional and post-transcriptional level. In particular, changes in the balance of pro- and anti-apoptotic factors determine whether cells die or survive.

The involvement of SR in physiological NMDAR activity and its crucial contribution to NMDAR overactivaction in many pathological settings has been widely documented. We, for the first time, have investigated whether the induction of the apoptotic program in a neuronal population—strongly dependent on NMDAR signaling for their survival and proper differentiation—induced a change in the enzymes related to D-serine metabolism, mainly SR (Esposito et al., 2012).

Cerebellar granule cell cultures are very useful for studying mechanisms involved in apoptosis and excitotoxic cell damage as they contain a relatively homogeneous population of neurons and are known to express different glutamate receptors. In a serum-based medium containing an inhibitor of mitosis and a high concentration of KCl (25 mM), CGNs can be maintained with high purity (95–98%). Under these conditions the proportion of nonneuronal cells (astroglia and interneurons) is very low (1–2%). CGNs undergo massive cell death when the depolarizing potassium concentration normally employed for cell culture is reduced from 25 (K25) to 5 mM (K5). This manipulation, experimentally compared to the surgical disconnection of the nerve afferents to CGN (Borsello et al., 2000) taking place either during embryogenesis or in various neurological diseases, activates an internal program of PCD in which biochemical and morphological elements of apoptosis and autophagy and UPS intersect and influence each other (Canu et al., 2000, 2005). This apoptotic cell death mimics the naturally occurring death of 20–30% of granule cells, a process important for matching the number of granule cells with Purkinje cells between the 3rd and 5th week postnatally after granule migration, which is known to require SR activation and D-serine release (Kim et al., 2005). Cell death evoked by K5 is associated with DNA fragmentation and caspase activation and requires both new RNA and protein synthesis (D'Mello et al., 1993): in fact, addition of protein or RNA synthesis inhibitors within the first 4 h of exposure to K5 prevents cell death and results in a complete recovery of the damaged DNA (D'Mello et al., 1993). However, the activation of the cell death program becomes irreversible in ≈50% of CGNs after 6 h of exposure to K5 (Nardi et al., 1997).

### Serine racemase is downregulated during the early phase of apoptosis

Time course studies have indicated that the level of SR protein is modulated very early during CGN apoptosis, before significant cell death. Interestingly, a biphasic expression pattern for SR protein was observed, with a drastic drop in protein levels by 50 and 70% at 6 and 24 h after apoptosis induction, respectively, followed by a second wave of increased expression in the late phase of apoptosis. Such SR changes were not observed in glial cells, which account for only 1% of the total cells in culture (Esposito et al., 2012). The finding that a decrease in SR protein in CGNs undergoing apoptosis was accompanied by a reduction in intracellular D-serine levels gives support to the recent report that D-serine is predominantly produced by neuronal cells (Ehmsen et al., 2013).

Interestingly, the decrease in SR protein was only reversed by UPS inhibitors, JNK inhibitors, application of NMDA, and/or D-serine. By contrast, inhibitors acting in the execution phase of apoptosis were unable to prevent it, suggesting that the decline in SR occurs in the commitment, transcription-dependent, phase (Esposito et al., 2012). Signaling pathways known to be involved in the control of neuronal survival are regulated during neuronal apoptosis at the transcriptional level and not only by post-translational mechanisms (Desagher et al., 2005). Similarly, SR decline is likely due to reduced transcription, as SR mRNA is rapidly downregulated after KCl deprivation via a pathway dependent on UPS and JNK kinase. Concerning the mechanism by which UPS and JNKs—two crucial players in CGN apoptosis (Canu et al., 2000; Harris et al., 2002)—regulate SR expression, we suggest that they likely act via mechanisms that could involve c-Jun. c-Jun, whose expression and phosphorylation increases rapidly after KCl withdrawal (Xifró et al., 2006), is known to induce transcription of SR in microglia cells treated with beta amyloid (Aβ) by JNK-dependent recruitment of AP-1 complex (b-Jun-c-Fos) on SR intron 1c (Wu and Barger, 2004). It is known that the AP-1 complex determines transactivation potential (Kaminska et al., 2000). Thus, we suggest that during neuronal apoptosis JNK should reduce SR transcription through an AP-1 complex comprising ATF-2 and c-Jun, given that c-Fos is rapidly downregulated during cerebellar apoptosis (Yuan et al., 2009). However, post-transcriptional mechanisms may also contribute to modulating SR expression. SR has a relatively short half-life and UPS regulates its turnover in a Golga3-modulated manner; see above (Dumin et al., 2006). It is conceivable that activation of UPS in the early phase of apoptosis may degrade SR as well (Canu et al., 2000). Further studies are needed to clarify the molecular mechanisms that govern SR regulation during apoptotic events. This has implications for SR downregulation under conditions characterized by NMDAR hypofunction and apoptotic events. For example, SR is downregulated in the nucleus accumbens of rats treated with cocaine (Curcio et al., 2013), which is known to be toxic for different cell types by increasing active caspase-3 and reactive oxygen species (Lepsch et al., 2009; Costa et al., 2013), and during aging (Mothet et al., 2006; Turpin et al., 2011; Billard, 2013), where neuronal cell loss has been attributed in part to increased NO production and high caspase activity (Akbulut et al., 2013).

### Why is serine racemase downregulated during the early phase of neuronal apoptosis?

The most obvious answer to this question is that the apoptotic program implies that neuronal sensitivity to glutamate is modulated and may thereby influence the mode of cell death, preventing excitotoxic necrosis and ensuring apoptosis. One possible mechanism by which apoptosis might modulate glutamate responses is by affecting glutamate receptor subunits. For example, in primary hippocampal neurons induced to undergo apoptosis by staurosporine, caspase-3-mediated degradation of AMPAR has been reported to be crucial for correctly driving neurons down the apoptotic pathway preventing necrosis (Glazner et al., 2000). In our model the situation is quite different since: (a) levels of both AMPAR and NMDAR subunits NR1, NR2A, and NR2B do not change after KCl deprivation (Canu, unpublished data); (b) level of SR is unchanged in CGNs induced to undergo apoptosis by staurosporine, suggesting that modulation of SR expression by apoptosis is stimuli-dependent (Canu, unpublished data); and (c) pharmacological inhibition of SR by phenazine-metosulphate and shRNA-mediated suppression of SR both exacerbate K5-death-inducing signaling.

Therefore, if apoptotic CGNs were decreasing the level of SR (and of D-serine) to prevent necrosis, then it would be expected that cell death would increase by adding D-serine to neurons undergoing apoptosis. Actually, adding D-serine (50–100 μM) to medium of CGNs undergoing apoptosis (K5) or overexpression of SR by adenovirus-mediated transduction does not increase cell death, but rather helps CGNs recover from apoptosis. Under both conditions, protection (which is NMDAR-dependent since it is blocked by the concomitant addition of 5,7-dichlorokynurenic acid, DCKA, a selective NMDAR antagonist acting at the glycine site) involves prevention of Akt and ERK1-2 dephosphorylation, JNK phosphorylation, and caspase activation and caspase-mediated cleavage of tau protein.

Thus, by decreasing the level of D-serine, apoptotic neurons avoid fully activating NMDAR and therefore stimulating survival molecules such as Akt and ERK1-2, whose kinase activities have been demonstrated to be critical in transmitting survival signals in this model of neuronal apoptosis (Shimoke et al., 1998; Chin et al., 2004). Moreover, the apoptotic program ensures the decrease in D-serine levels through the opposite regulation of SR and DAAO. By microarray assay, we found that the DAAO gene is significantly (2-fold) upregulated and that its activity is increased by +82, +170, and +90% at 1, 3, and 6 h of apoptosis, respectively, compared to control nonapoptotic neurons (Esposito et al., 2012). Moreover, the DAAO inhibitor sodium benzoate is able to reduce D-serine decline and also increase survival (Canu, unpublished data). The opposite regulation of the two main D-serine metabolic enzymes has been reported under other experimental conditions. DAAO and SR, when present in the same cells, do not work in isolation; e.g., their activities are regulated in opposite directions by nitric oxide (Shoji et al., 2006a,b) or by cocaine (Curcio et al., 2013) to tightly regulate D-serine level in glioma cells and in the nucleus accumbens, respectively.

Divalent cations and phosphorylation at Thr71 by proline-directed kinases, such as ERK1-2 (Foltyn et al., 2010), are necessary for complete racemase activity. It is noteworthy that the apoptotic process is associated with an immediate decrease in the levels of intracellular calcium (Galli et al., 1995) and activated ERK1-2 (Chin et al., 2004): it is thus reasonable to suppose that apoptosis may also modulate SR activity. Indirect evidence in support of this hypothesis is the finding that L-serine (1–2 mM) can prevent cell death without activating NMDAR, suggesting that it is not converted in D-serine in CGNs undergoing apoptosis (Esposito et al., 2012).

### Serine racemase level is increased in the late phase of apoptosis and contributes to channel neurons toward a necrotic phenotype

At the biochemical level, one of the most impressive SR changes is the increase in expression and function at late stages of apoptosis. Western blot analysis of cell extracts from apoptotic neurons showed that SR expression after 48 h was twice that observed at 24 h of apoptosis. SR increase in dying neurons was also confirmed by immunofluorescence analysis. Increased SR expression was positively accompanied by a marked increase in the level of D-serine in the medium of CGN undergoning apoptosis (K5) (0.5 ± 0.13 μM in control neurons to 10.3 ± 0.7 μM in apoptotic neurons). What are the functional consequences of SR-D-serine pathway increase in the late phases of apoptosis? It might be the last, but futile attempt to save neurons from death, providing new survival signals through the NMDAR. However, the finding that phenazine metasulfate inhibitor of SR activity—added 48 h after inducing apoptosis—reduces the D-serine content and saves these remaining neurons from death suggests that this late SR-D-serine increase is toxic to CGNs. Indeed, we found that toxicity is blocked by MK801, a noncompetitive NMDAR antagonist, thus demonstrating that this last wave of cell death is NMDAR-mediated and of the necrotic or oncotic type. This term is used to describe cell death that is accompanied by cell swelling and, eventually, disruption of the cell membrane. The loss of cell membrane integrity results in the release of the cytoplasmic content. Accordingly, lactate dehydrogenase (LDH) is increased in the medium of apoptotic neurons in the late phase of apoptosis. LDH increase is prevented by phenazine metasulfate and MK801, confirming that it is triggered by SR increase. Evidence indicates that necrosis and apoptosis represent morphological expressions of a shared biochemical network described as the “apoptosis-necrosis continuum” (Zeiss, 2003). Factors that convert an ongoing apoptotic process into a necrotic process include a decrease in the availability of caspases and intracellular ATP (Leist et al., 1999; Denecker et al., 2001). Secondary necrosis has been reported to occur as a consequence of: (a) intracellular Ca^2+^ overload due to caspase-dependent inactivation of plasma membrane calcium pumps in CGN undergoing apoptosis by a low dose of glutamate (Schwab et al., 2002); (b) NR2B-mediated cell death due to accumulation of caspase-3- and calpain-generated N-terminal tau fragment in CGNs undergoing apoptosis by KCl deprivation (Amadoro et al., 2004, 2006) and (c) proteasome inhibition (Canu and Calissano, 2006).

Regarding the mechanisms that cause SR to accumulate in the late phase of apoptosis, we found that increased SR expression was not dependent on the increased transcription or increased stability of SR mRNA. As stated above, SR turnover is regulated by proteasomes in a Golga3-dependent manner. During CGN apoptosis, UPS and many Golgi-localized proteins became part of a generalized cellular failure that affects the major activities of the apoptotic neurons after caspase activation (Canu et al., 2000; Mancini et al., 2000). Therefore, we can envisage that loss of Golga3 and UPS impairment causes SR accumulation and death of neurons in the late stages of apoptosis. This hypothesis is supported by the finding that cell death caused by pharmacological inhibition of UPS is prevented by the SR inhibitor phenazine-metasulfate (Canu et al., unpublished observation). The involvement of NMDAR in cell death induced by UPS inhibition is under investigation.

## Serine racemase and neurological disorders

Proteins of glutamatergic NMDAR signaling pathways have been studied as targets for intervention in a variety of neuropathological conditions. D-Serine is now recognized to be involved in controlling the extent of NMDAR activation and neurotoxic insults observed in many central nervous disorders (Danysz and Parsons, 1998).

### Schizophrenia

Schizophrenia is a long-term mental health condition characterized by a dissociation of various components of the personality. The etiology of schizophrenia involves a combination of genetic, neurodevelopmental, social, and environmental factors. The observation that NMDAR antagonists such as phencyclidine and ketamine were able to induce a psychotomimetic state resembling schizophrenia in human subjects prompted the hypothesis that NMDAR hypofunction was implicated in the pathophysiology of schizophrenia. A deficiency in endogenous D-serine availability has been proposed to be one of the underlying causes of NMDAR hypofunction.

Consistent with this hypothesis, several studies have revealed low levels of SR and low concentrations of D-serine in the serum and cerebrospinal fluid in subjects with schizophrenia. Moreover, D-serine reduces negative symptoms and improved cognition in patients with chronic schizophrenia treated with antipsychotics, reviewed in Van Horn et al. (2013).

Genetic association studies have implicated genes coding for enzymes associated with D-serine metabolism in schizophrenia. These include single-nucleotide polymorphism variants of SR, DAAO, and the DAAO interacting protein pLG72, reviewed in Labrie and Roder (2010) and Pollegioni and Sacchi (2010). Remarkably, disrupted in schizophrenia-1 (DISC1), a protein involved in the pathophysiology of schizophrenia and other psychiatric disorders, directly binds SR, protecting it from ubiquitin-mediated degradation (Ma et al., 2013). Mutant DISC1 fails to bind SR, facilitating ubiquitination and degradation of SR and a decrease in D-serine level production. PICK1, another SR interactor, has also been linked to schizophrenia (Fujii et al., 2006). Moreover, neonatal inactivation of SR by phenazine methasulfate causes behavioral abnormalities in later life. Thus, multiple risk pathways for schizophrenia may converge on SR depletion (Balu et al., 2013). This hypothesis is supported by the finding that anatomical deficits in schizophrenia patients—such as reduced hippocampal volume, decreased dendritic spine density, altered neuroplasticity likely due to an increase in apoptotic events (Catts and Weickert, 2012)—are also shared by SR-null mutant mouse—SR(–/–), a mouse model of NMDAR hypofunction (Balu et al., 2013). It is worth pointing out, however, that the morphological deficits found in schizophrenia patients are the result of a combination of multiple factors, among them the antipsychotic drugs received. This could account, for example, for the increased expression of SR found in patients suffering from schizophrenia and receiving antipsychotic drugs (Verrall et al., 2007) such as clozapine, which it is known to increase D-serine release (Tanahashi et al., 2012).

### Alzheimer disease (AD)

AD is a progressive disease in which dementia symptoms gradually worsen over a number of years. In its early stages, memory loss is mild, but with late-stage Alzheimer's, individuals lose their ability to carry on a conversation and respond to their environment. Both reduced expression of NMDAR (Jansen et al., 1990; Sze et al., 2001) and reduced levels of D-serine have been implicated in NMDAR hypofunction in the early phase of AD (Snyder and Kim, 2000; Wolosker et al., 2002). D-Cycloserine, a partial agonist at the glycine site of NMDAR, and the DAAO inhibitor sodium benzoate were reported in some clinical studies to activate the NMDAR in brains of AD patients (Chessell et al., 1991; Lin et al., 2013) and improve their cognitive function (Lin et al., 2013). Despite these findings, NMDAR activity is supposed to be increased in moderate to severe AD as memantine, a NMDAR antagonist, is recommended as an option for managing moderate and severe AD for patients who cannot be treated with acetylcholinesterase inhibitor (AChEI) (Wilkinson et al., 2013). The NMDAR activity in the early and late phase of AD may be related to different regulation of SR expression and may be linked to different modes of cell death. Indeed, in the early phase of AD, caspase activity is increased (Ramcharitar et al., 2013) while in late AD, proteasome impairment and other caspase-independent mechanisms of cell death occur. The two main hallmarks of AD, Aβ and tau protein, may alone or in combination perturb the distribution, the density, and the sensitivity of NMDAR (Ittner et al., 2010). Nothing is known about the effect of tau-based pathology of the SR/D-serine pathway. By contrast, Aβ is reported to induce the release of D-serine and glutamate from cultured microglia, but not from cultured hippocampal neurons. In particular, Aβ increases microglial SR transcription (Wu et al., 2004) through JNK-dependent recruitment of AP-1 complex to SR promoter (Wu and Barger, 2004).

Conditioned medium from Aβ-treated microglia is toxic to cultured hippocampal neurons. Toxicity, which tends to manifest as necrosis, is prevented by the NMDAR glycine site antagonist 5,7-dicholorokynurenic acid and by enzymatic degradation of D-amino acids by DAAO. SR mRNA levels were reported to be elevated in microglia cultures as well as in AD hippocampus, likely suggesting that it contributes to excitotoxic neuronal death in severe AD (Wu and Barger, 2004; Wu et al., 2004), as supported by recent findings that Aβ-induced neurotoxicity is attenuated in SR knockout mice (Inoue et al., 2008). The form of pathogenic amyloid (aggregated, oligomeric, or soluble Aβ) responsible of increased SR expression in AD and why the effect is specific only for microglia and not for neurons remain to be established. The following question then arises: is SR modulated in neurons undergoing apoptosis by Aβ (Troy et al., 2000)?

### Amyotrophic lateral sclerosis (ALS)

ALS is the most common neuromuscular disease characterized by dysfunction and death of both upper and lower motoneurons, leading to fatal paralysis. The pathogenesis of this disease and the reasons for the selective pattern of neurodegeneration remain uncertain, but mechanisms currently proposed for cell death in ALS include excitotoxicity and oxidative stress. Patients with ALS have impaired glutamate transporters and D-serine levels in the spinal cord and motor cortex that could account for NMDAR overactivation (Sasabe et al., 2012). SR and D-serine are involved in both the pre-symptomatic and progressive phases of ALS in G93A Cu, Zn-superoxide dismutase (SOD-1) mice, the standard model of ALS transgenic mice, suggesting a link between mutant SOD-1 and D-serine increase in ALS (Sasabe et al., 2012).

Levels of D-serine and SR in the spinal cords of SOD-1 mutant mice are progressively elevated, mainly in glia cells as a consequence of pathological activation (Sasabe et al., 2012). ALS mice with SR deletions show earlier symptom onset, but survive longer, in an SR-dependent manner. Paradoxically, administration of D-serine to ALS mice lowers spinal cord levels of D-serine, leading to changes in onset and survival that are very similar to SR deletion (Thompson et al., 2012). The mechanism by which SOD-1 mutation increases SR expression and D-serine level is not known.

Mutant isoform of the SOD1 protein impairs UPS function (Kabashi et al., 2004)—likely by decreasing constitutive proteasome—and increases SR expression when overexpressed in microglia cells (Sasabe et al., 2012). Given that both SR and pLG72, the DAAO-interacting partner (Sacchi et al., 2008), are regulated by the proteasome system (Dumin et al., 2006; Cappelletti et al., 2014), it is tempting to speculate that proteasome impairment in SOD-1 mutated mice increases both SR and pLG72 protein expression, conditions which result in high levels of D-serine and consequently in NMDAR overactivation. Alternatively, increased expression of SR may result from increased transcription in microglia activated by cytokines, which are strongly induced in spinal cord of G93A-SOD1 mice (Hensley et al., 2002)

Other neurodegenerative diseases, such as PD and HD, have been associated with altered NMDAR activity. It has been suggested that NMDAR stimulation, accomplished through allosteric modulation via the glycine modulatory site may be beneficial in late-phase PD. Indeed, preliminary findings suggest that D-serine treatment, in addition to usual medications, may be beneficial in PD, resulting in increased D-serine serum levels and in improving motor deficits (Gelfin et al., 2012; Heresco-Levy et al., 2013). However, little is known about how and if D-serine content or SR and DAAO activities are modulated in PD. Similarly, our knowledge on SR/D-serine pathway is more scarce or absent for HD (a neurodegenerative genetic disorder that affects muscle coordination and leads to cognitive decline and psychiatric problems), where cell death has been linked to abnormally enhanced NMDAR activity (Marco et al., 2013).

### Epilepsy

Temporal lobe epilepsy is associated with various cognitive changes, but the mechanisms underlying these changes are unknown. It has been recently reported that D-serine deficiency is important in the amnestic symptoms of temporal lobe epilepsy (Klatte et al., 2013). Indeed, this condition in rats is associated with a reduction in D-serine levels in the central nervous system, which leads to a desaturation of the coagonist binding site of synaptic and extrasynaptic NMDARs. NMDAR desaturation causes a deficit in hippocampal LTP, which can be rescued with exogenously supplied D-serine. For example, added, exogenous D-serine improved spatial learning in epileptic animals (Klatte et al., 2013). It is worthy of note that D-serine decline is linked to decreased expression of SR—at both mRNA and protein levels—in the hippocampal CA1 region and to increased expression of mRNA for DAAO (Klatte et al., 2013). This finding is similar to the changes observed in neuronal apoptosis (Esposito et al., 2012), in aging (Mothet et al., 2006; Turpin et al., 2011), and in the nucleus accumbes of cocaine-treated rats (Curcio et al., 2013), suggesting that apoptotic mechanisms may determine SR/D-serine pathway changes in epilepsy. This result is supported by findings that neuronal death, which has been implicated as a causal factor in epileptogenesis, is controlled by apoptosis-associated molecular mechanisms, reviewed in Henshall and Engel (2013).

### Stroke and ischemia

Neuronal damage resulting from overactivation of NMDA receptors contributes to acute disorders such as ischemia and trauma that are prevented by agents blocking NMDAR activity, such as memantine. High levels of D-serine have been observed in simulated (oxygen/glucose deprivation, OGD) ischemia models, causing neuronal death (Katsuki et al., 2004). Evidence for a central role of D-serine synthesis from L-serine by SR during OGD was given in SR knockout mice, displaying 90% reduced D-serine concentrations and decreased neurotoxicity and dramatically diminished infarct volume after middle cerebral artery occlusion (MCAO) (Mustafa et al., 2010). These changes are accompanied by marked declines in NO formation and nitrosylation of its targets. Interestingly the pronounced reduction in infarct volume of SR knockout animals following MCAO is comparable to protection against stroke damage associated with pharmacologic blockade of NMDA receptors. Since no increase in SR expression (but rather a decrease in SR expression was observed during the first 12 h of ischemic insult) or decrease in DAAO expression was observed in different models of ischemia, it is reasonable to suppose that increased levels of D-serine may result from increased SR activity (likely as a consequence of phosphorylation, changes in intracellular distribution, and/or change in binding partners), decreased DAAO activity, altered shuttling of D-serine across membranes, and or altered NR1 expression and sensitivity.

## Conclusions

Extensive research in the past 30 years has shed molecular light onto the mechanisms linking NMDAR physiology and pathophysiology to neuronal survival/plasticity and to neuronal diseases (such as acute stroke, neuropsichiatric disease, and neurodegenerative disorders), respectively. Although we have already discovered several important targets that play important roles in mediating NMDAR activity, the present challenge is to develop novel compounds that will be selective for the excitotoxic or pro-survival effects of NMDAR stimulation.

The development of such compounds must deeply rely on the complex mechanisms and pathways of neuronal demise (triggered by lack of survival signals or by toxic insults), which, as widely stated in this review, often implies the interplay and shift of different modes of cell death (e.g., apoptosis/necrosis or necrosis/apoptosis shift) (Jellinger, 2001; Andorfer et al., 2005; Canu et al., 2005). On the other hand, our understanding of the complexity of the mechanisms that governs cell death is also currently evolving with the identification of novel key regulators of this process. In the present review we highlighted new findings showing the importance of the dual and opposite role of the SR/D-serine pathway in the fine regulation of neuronal apoptosis and the apoptosis/necrosis shift (Esposito et al., 2012) both in “*in vitro*” and “*in vivo*” conditions. Many questions still remain regarding how the cell death process influences—via transcriptional and/or post-transcriptional (phosphorylation) mechanisms—SR activity and also other components of the D-serine pathway. The answer to these questions, and in particular to post-transcriptional mechanisms involved in SR activity during different stages of apoptosis (such as phosphorylation, nitrosylation, etc.), might have implications for a deeper understanding of the conformational plasticity underlying SR response to physiological effectors and to potential therapeutic agents.

Moreover, special attention should be paid in the future to the involvement and modulation of other components of the D-serine pathway (e.g., DAAO, D- and L-serine transporters) in the cell death process and NMDAR activity, in light of recent findings on the contribution of D-serine transporters (through the release of D-serine) to synaptic NMDAR activity (Rosenberg et al., 2013). Similarly, the role of L-serine transporters in the supply of L-serine to neurons by astrocytes for the synthesis of D-serine by serine racemase (Ehmsen et al., 2013) is also important.

### Conflict of interest statement

The authors declare that the research was conducted in the absence of any commercial or financial relationships that could be construed as a potential conflict of interest.
